# Supplementary material for: Intestinal Microbiota in Postmenopausal Breast Cancer Patients and Controls
Source: Cancers (Basel). 2021 Dec 9;13(24):6200. doi: 10.3390/cancers13246200 (PMC8699039; doi:10.3390/cancers13246200)
Supplement: Supplementary file 1 [file cancers-13-06200-s001.zip › 1. Tables supplement BC vs C 2021_10_14.pdf]

Supplementary tables

Table S1: Clinical characteristics of the study population – Breast cancer group

**Baseline characteristics**	**Breast cancer** ***n*=81**	**Neoadjuvant** ***n*=18**	**Adjuvant** ***n*=63**	***p*-value**
Chemotherapy past - No. (%)				
No	78 (96)	18 (100)	60 (95)	0.345
Yes	3 (4)	0 (0)	3 (5)	
Time since last chemo – Weeks				
Mean (SD)	196 (278)	-	196 (278)	
Min-Max	22-517		22-517	
Radiotherapy received - No. (%)				
No	16 (20)	3 (17)	13 (21)	**<0.001**
Yes	65 (80)	15 (83)	50 (79)	
Timing radiotherapy - No. (%)*				
Before therapy	18 (22)	0 (0)	18 (29)	
During therapy	19 (24)	0 (0)	19 (30)	**<0.001**
After therapy	28 (35)	15 (83)	13 (21)	
Unknown	16 (20)	3 (17)	13 (21)	

* Percentages do not add up to 100% due to rounding.

Table S2: Microbial richness and diversity measures

**Microbial richness and diversity**	**Total** ***N*=148**	**Breast cancer** ***n*=81**	**Controls** ***n*=67**	***p*-value**
Observed richness				
Median (IQR)	234 (79)	234 (78)	233 (85)	0.561
25-75%	185-264	184-261	188-273	
Shannon index				
Mean (SD)	3.95 (0.36)	3.92 (0.38)	4.00 (0.33)	0.207
Min - Max	3.04-4.64	3.04-4.64	3.33-4.60	

Table S3: Microbial richness and diversity measures

**Microbial richness and diversity**	**Controls** ***n*=67**	**Neoadjuvant** ***n*=18**	**Adjuvant** ***n*=63**	***p*-value**
Observed richness				
Median (IQR)	233 (85)	248 (62)	230 (76)	0.288
25-75%	188-273	205-267	181-257	
Shannon index				
Mean (SD)	4.00 (0.33)	4.07 (0.44)	3.88 (0.36)	0.057
Min - Max	3.33-4.60	3.04-4.64	3.04-4.54	

Table S4: Differential abundant taxa on family and genus level of patients scheduled for neoadjuvant treatment, adjuvant treatment, and controls

**Taxa**	**Controls** ***n*=67**	**Neoadjuvant** ***n*=18**	**Adjuvant** ***n*=63**	***p*-value**
Veillonellaceae				
Median (IQR)	1.63 (2.87)	0.48 (1.2)	2.53 (2.15)	**0.004**
25%-75%	0.00-2.87	0.00-1.20	0.90-3.05	
Dialister				
Median (IQR)	0.00 (2.85)	0.00 (0.00)	2.45 (3.05)	**0.003**
25%-75%	0.00-2.85	0.00-0.00	0.00-3.05	

log^{10} abundance of taxa are presented.

Table S5: Adjusted *p*-values of pairwise comparison of the differential abundant taxa of patients scheduled for neoadjuvant treatment (NA), adjuvant treatment (A), and controls (C)

**Taxa**	**C vs NA**	**C vs A**	**NA vs A**
Veillonellaceae	0.116	0.226	**0.004**
Dialister	0.089	0.234	**0.003**

P-values have been adjusted by the Bonferroni correction for multiple testing.
log^{10} abundance of taxa are presented.
