# Supplementary material for: Intestinal Microbiota in Postmenopausal Breast Cancer Patients and Controls
Source: Cancers (Basel). 2021 Dec 9;13(24):6200. doi: 10.3390/cancers13246200 (PMC8699039; doi:10.3390/cancers13246200)
Supplement: Supplementary file 1 [file cancers-13-06200-s001.zip › 2. Methods supplement BC vs C 2021_10_14.pdf]

Supplementary material and methods

Faecal microbiota analyses

In order to extract metagenomic DNA, 250 mg of the frozen faecal samples were homogenised in phosphate buffered saline (PBS) and were centrifuged for 1 minute at 900 rpm. For cell lysis, a combination of chemical, mechanical and thermal disruption was used. A lysis buffer containing 1M Tris-HCl, 0.5M EDTA, 5M sterile NaCl and SDS (final concentration 4%) was filled into bead tubes of the Ambion MagMax^{TM} Total Nucleic Acid Isolation Kit *(Thermo Fisher Scientific)* and mixed with 175 µl supernatant of faeces in PBS. Mechanical disruption consisted of a bead-beating procedure using the Fastprep™ Homogenizer *(*5,5 ms for 3x1 min; resting 1 min in between, *MP Biomedicals)*. Samples were subsequently incubated for 15 minutes at 95°C with gentle shaking. After centrifugation for five minutes at 11000 rpm, supernatant was filled in an Eppendorf tube. Afterwards, a second round of bead beating and incubation was performed and supernatants were pooled and stored at -20°C until further analysis. 200 µl of the supernatants were introduced into a KingFisher 96-wells deep well plate *(Thermo Fisher Scientific)*, together with bead mix of the Ambion MagMax^{TM} Total Nucleic Acid Isolation Kit (*Thermo Fisher Scientific*), isopropanol, and lysis buffer. Other plates were filled with wash buffers, elution buffer (+RNAse), and 96-tips for DW magnets *(Thermo Fisher Scientific)*. Afterwards, the prepared plates were introduced into the KingFisher system and the DNA isolation was performed according to the manufacturer’s standard protocol *(Thermo Fisher Scientific).* After removal of the plates from the system, the plate containing purified nucleic acids was incubated for 15 minutes at 37°C for degradation of RNA.

Subsequently, the V4 hypervariable region of the 16S rRNA gene was amplified in triplicate using the 515F/806R barcoded primer pair described previously [1]. Pooled amplicons from the triplicate reactions were purified using AMPure XP purification beads (Agencourt) according to the manufacturer's instructions and eluted in 25 µl 1 × low TE (10 mM Tris-HCl, 0.1 mM EDTA, pH 8.0). Quantification of amplicons was subsequently performed by the Quant-iT PicoGreen dsDNA reagent kit (Invitrogen) using a Victor3 Multilabel Counter (*Perkin Elmer*). Amplicons were mixed in equimolar concentrations to ensure equal representation of each sample and sequenced on an Illumina MiSeq instrument (MiSeq Reagent Kit v3, 2 × 250 cycles, 10% PhiX) to generate paired-end reads of 250 bases (~25.000 reads/sample) [2].

The pre-processing of sequencing data, using an in-house pipeline based upon DADA2 (R version 4.0.3) [3], consisted of the following steps: reads filtering, identification of sequencing errors, dereplication, and removal of chimeric sequences. In order to assign taxonomy, DECIPHER[4] was used to annotate to the genus level. Data were expressed as amplicon sequence variants (ASVs). Decontam was used with the 'either' setting, which combines the two statistical methods prevalence and frequency for the identification of contamination in marker-gene and metagenomics data [5]. Contaminated ASVs identified by decontam were filtered out together with ASVs presented in less than 5% of all samples and a total abundance of less than 0.001%. A total of 816 ASVs were maintained for downstream analysis. The final file was saved in the phyloseq format [6].

References

[1] Caporaso, J. G., Lauber, C. L., Walters, W. A., Berg-Lyons, D., Huntley, J., Fierer, N., Owens, S. M., Betley, J., Fraser, L., Bauer, M., Gormley, N., Gilbert, J. A., Smith, G., and Knight, R. (2012) Ultra-high-throughput microbial community analysis on the Illumina HiSeq and MiSeq platforms, *ISME J 6*, 1621-1624.

[2] Galazzo, G., Tedjo, D. I., Wintjens, D. S. J., Savelkoul, P. H. M., Masclee, A. A. M., Bodelier, A. G. L., Pierik, M. J., Jonkers, D., and Penders, J. (2019) Faecal Microbiota Dynamics and their Relation to Disease Course in Crohn's Disease, *J Crohns Colitis 13*, 1273-1282.

[3] Callahan, B. J., McMurdie, P. J., Rosen, M. J., Han, A. W., Johnson, A. J., and Holmes, S. P. (2016) DADA2: High-resolution sample inference from Illumina amplicon data, *Nat Methods 13*, 581-583.

[4] Wright, E. S. (2016) Using DECIPHER v2.0 to Analyze Big Biological Sequence Data in R, *The R Journal 8(1)*, 352-359.

[5] Davis, N. M., Proctor, D. M., Holmes, S. P., Relman, D. A., and Callahan, B. J. (2018) Simple statistical identification and removal of contaminant sequences in marker-gene and metagenomics data, *Microbiome 6*, 226.

[6] McMurdie, P. J., and Holmes, S. (2013) phyloseq: an R package for reproducible interactive analysis and graphics of microbiome census data, *PLoS One 8*, e61217.
